# Role of Cytokines in Thymus- Versus Peripherally Derived-Regulatory T Cell Differentiation and Function

**DOI:** 10.3389/fimmu.2013.00155

**Published:** 2013-06-19

**Authors:** Jérémie David Goldstein, Louis Pérol, Bruno Zaragoza, Audrey Baeyens, Gilles Marodon, Eliane Piaggio

**Affiliations:** ^1^Université Pierre et Marie Curie Univ Paris 06, INSERM U959, Paris, France; ^2^Centre National de la Recherche Scientifique, UMR 7211, Paris, France; ^3^Institut National de la Santé et de la Recherche Médicale (INSERM), U959, Immunology-Immunopathology-Immunotherapy (I3), Paris, France; ^4^INSERM U932, Paris, France; ^5^Section Recherche, Institut Curie, Paris, France; ^6^INSERM Center of Clinical Investigation (CBT507 IGR-Curie), Paris, France

**Keywords:** tTreg, pTreg, IL-2, TNF-α, TGF-β, regulatory T cells, Foxp3

## Abstract

CD4^+^CD25^+^Foxp3^+^ regulatory T cells (Tregs) are essential players in the control of immune responses. Recently, accordingly to their origin, two main subsets of Tregs have been described: thymus-derived Tregs (tTregs) and peripherally derived Tregs (pTregs). Numerous signaling pathways including the IL-2/STAT5 or the TGF-β/Smad3 pathways play a crucial role in segregating the two lineages. Here, we review some of the information existing on the distinct requirements of IL-2, TGF-β, and TNF-α three major cytokines involved in tTreg and pTreg generation, homeostasis and function. Today it is clear that signaling via the IL-2Rβ chain (CD122) common to IL-2 and IL-15 is required for proper differentiation of tTregs and for tTreg and pTreg survival in the periphery. This notion has led to the development of promising therapeutic strategies based on low-dose IL-2 administration to boost the patients’ own Treg compartment and dampen autoimmunity and inflammation. Also, solid evidence points to TGF-β as the master regulator of pTreg differentiation and homeostasis. However, therapeutic administration of TGF-β is difficult to implement due to toxicity and safety issues. Knowledge on the role of TNF-α on the biology of Tregs is fragmentary and inconsistent between mice and humans. Moreover, emerging results from the clinical use of TNF-α inhibitors indicate that part of their anti-inflammatory effect may be dependent on their action on Tregs. Given the profusion of clinical trials testing cytokine administration or blocking to modulate inflammatory diseases, a better knowledge of the effects of cytokines on tTregs and pTregs biology is necessary to improve the efficiency of these immunotherapies.

Thymus-derived Tregs (tTregs), which emerge from the thymus as a distinct lineage, and peripherally derived Tregs (pTregs), which are generated outside the thymus from CD4^+^CD25^−^ T cell precursors under particular conditions of stimulation, present great similarities, and differences. They are both defined by the expression of the transcription factor Foxp3, widely recognized as the master regulator of Treg fate. This factor, expressed quite specifically by Tregs (Fontenot et al., 2003; Hori et al., 2003) is required for their suppressive function both *in vitro* and *in vivo* (Fontenot et al., 2003, 2005a; Hori et al., 2003). But Tregs’ specific genetic signature is only partially dependent on Foxp3 (Sugimoto et al., 2006; Gavin et al., 2007; Hill et al., 2007; Lin et al., 2007; Ohkura et al., 2012). And, in order to acquire this exclusive signature and mature into a stable lineage, both tTregs and pTregs will go through a process of “education” in several steps and different localizations. Here, we will describe the role of cytokines during this process. The role of the TCR in Treg development and of cytokines in Treg effector mechanisms have been the subject of recent excellent reviews (Vignali et al., 2008; Ohkura et al., 2013) and will not be extensively discussed here.

## Thymic Derived Treg Cells

tTregs have been defined by the constitutive expression of the high affinity IL-2Rα chain, CD25 (Sakaguchi et al., 1995). They are selected in the thymus based on their recognition of self-antigens by a TCR of high avidity (Jordan et al., 2001) and represent an important fraction of the total Tregs found in periphery (Hsieh et al., 2006; Josefowicz et al., 2012a). Removal of the thymus early after birth leads to various autoimmune symptoms (Itoh et al., 1999), suggesting that tTregs participate in the continuous prevention of spontaneous autoimmunity.

### IL-2 regulates both thymic development and peripheral homeostasis of tTreg

**Figure 1:**
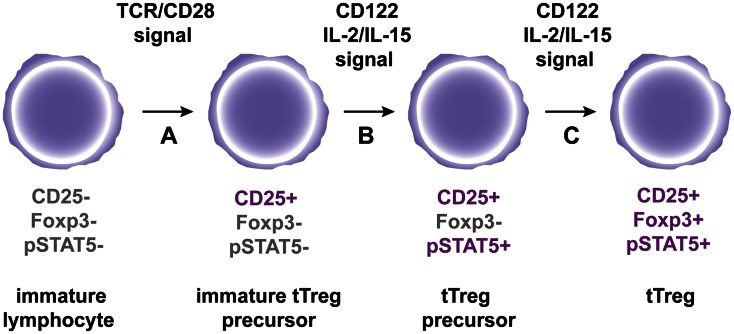
**A hypothetic model for tTreg differentiation in the thymus**. **(A)** Immature CD4^+^ thymocytes are engaged by strong agonist TCR/co-stimulatory signals, which results in the expression of the IL2R alpha chain CD25. **(B)** Subsequent interaction of immature tTreg precursors with CD122 signaling cytokines IL-2 and IL-15 leads to STAT5 phosphorylation to generate tTreg precursors. **(C)** Following continuous engagement of CD122, Foxp3 expression is induced in tTreg precursors to generate fully mature tTregs.

The role of IL-2 in tTreg differentiation and homeostasis has been extensively studied. Early work showed that mice deficient for IL-2 or CD25 were profoundly deprived of Tregs in the periphery but not in the thymus (D’Cruz and Klein, 2005; Fontenot et al., 2005b), suggesting that IL-2 was mandatory for Treg homeostasis in the periphery but not for thymic generation. However, mice doubly deficient for IL-2 and IL-15, or for the IL-2Rβ chain (CD122) common to IL-2 and IL-15, present a quasi-complete depletion of thymic Treg cells (Burchill et al., 2007; Soper et al., 2007). Consequently, the CD122 signaling is mandatory for proper differentiation of tTregs. At the molecular level, binding of the CD122 signaling intermediate STAT5 to the conserved non-coding DNA sequence 2 (CNS2) element at the *Foxp3* locus is required for optimal Foxp3 expression (Zorn et al., 2006; Burchill et al., 2007; Yao et al., 2007; Mouly et al., 2010) (for a recent review on the role of cytokine-induced transcription factors regulating Foxp3 expression, see Merkenschlager and von Boehmer, 2010). Demethylation of the CNS2 is the hallmark of stable Tregs but the role played by IL-2 in this process appears minimal since IL-2 cannot drive demethylation of the CNS2 in CD25^*hi*^Foxp3^−^ tTreg precursor if applied in the absence of TCR signals (Toker et al., 2013). Indeed, this precursor population expresses Foxp3 *in vitro* upon IL-2 stimulation without requirement for additional TCR signaling (Lio and Hsieh, 2008). Thus, a two-step model for Treg differentiation has been proposed in which TCR/CD28 signals first induce the differentiation of this precursor with enhanced sensitivity to IL-2/IL-15, followed by direct Foxp3 induction by IL-2/IL-15 signaling in a STAT5-dependent TCR-independent manner (Burchill et al., 2008; Lio and Hsieh, 2008). However, we believe that this two-step model is incomplete. Indeed, we have recently demonstrated that a minority of tTreg precursors expressed pSTAT5 *ex vivo* in unmanipulated neonates and we propose that this subset might be the direct precursors of pSTAT5^+^ CD25^+^Foxp3^+^ tTregs (Figure 1) (Goldstein et al., 2011). Nevertheless, not all Foxp3^+^ T cells express pSTAT5, suggesting either that Foxp3 expression can be maintained without continuous STAT5 phosphorylation or that differentiation of tTreg may proceed through a STAT5-independent pathway. Thus, the exact mechanism by which CD122 signaling controls the generation of tTregs remains to be determined.

Outside the thymus, the role of IL-2 on Treg homeostasis is widely accepted: no IL-2, no functional tTregs in the periphery. Results obtained in mice deficient for IL-2 (Sadlack et al., 1993, 1995) or its receptor (Suzuki et al., 1995; Willerford et al., 1995), which develop extensive lymphadenopathy and die of systemic auto-immunity early after birth, extended the role of IL-2 from a “T Cell Growth Factor” (Smith et al., 1980) to “the gatekeeper of immunological tolerance”. The autoimmunity observed in IL-2/IL-2R KO mice (whether CD25, CD122, or CD132 KO, the different components of the IL-2R) is associated with a profound deficit in Treg numbers and function, suggesting that tTregs generated in the absence of IL-2/IL-2R signaling cannot survive in the periphery. This view has been challenged by others, who reported the presence of Foxp3^+^ cells in the periphery of IL-2 KO mice (Liston et al., 2007). Noteworthy is the lack of autoimmunity in this later study. Thus, the association between the lack of IL-2, Treg deficit, and autoimmune manifestations cannot be always made. In further support of an important role for IL-2 in Treg survival, *in vivo* neutralization of IL-2 by the injection of an anti-IL-2 antibody results in the rapid depletion of Tregs and in the appearance of systemic, albeit limited, autoimmunity (Setoguchi et al., 2005). IL-2 is required for the maintenance of Foxp3 protein and mRNA expression both *in vitro* and *in vivo* (Setoguchi et al., 2005; Murawski et al., 2006; Passerini et al., 2008; Rubtsov et al., 2010). Furthermore, Tregs are crucially dependent on paracrine IL-2 production by effector T cells (Teffs), as they cannot produce IL-2 due to direct Foxp3-mediated repression of IL-2 transcription (Wu et al., 2006). Worth mentioning, the number of Tregs is indexed to the number of IL-2 producing Teffs (Almeida et al., 2006). In addition, polymorphisms in IL-2, CD25, or downstream adaptors genes are associated with impaired Treg numbers or function and higher susceptibility to autoimmunity (Bottini et al., 2004; Vella et al., 2005; Todd et al., 2007; Yamanouchi et al., 2007; Liston et al., 2008; Sgouroudis et al., 2008, 2011). Indeed, we and others have shown that IL-2 administration to mice (Tang et al., 2008; Wilson et al., 2008; Webster et al., 2009; Grinberg-Bleyer et al., 2010a; Dinh et al., 2012) and humans (Koreth et al., 2011; Saadoun et al., 2011) increases Treg numbers, reinforces their suppressive function, and protects from chronic inflammation. Indeed, low-dose IL-2 administration to pre-diabetic NOD mice which prevents disease development, increases Treg proportions specifically in the pancreas and these IL-2 expanded Tregs express higher levels of Bcl-2, CD25, and Foxp3, suggestive of increased resistance to apoptosis and higher activation (Tang et al., 2008). Furthermore, IL-2 administration to new onset diabetic NOD mice which reverts hyperglycemia, does not significantly increase Treg frequencies, yet Tregs express higher levels of molecules associated to Treg function, such as CD25, Foxp3, GITR, and ICOS and there is a decreased production of IFN-γ by pancreas infiltrating CD8^+^ T cells (Grinberg-Bleyer et al., 2010a). These results suggest that IL-2-boosted Tregs may have an improved suppressive function. The demonstration that IL-2 is critical for Treg biology has opened new perspectives for the treatment of inflammatory diseases.

### A more uncertain role for TGF-β on tTreg development and function

The role of TGF-β during tTreg differentiation is controversial. Mice deficient for TGF-β (Shull et al., 1992; Marie et al., 2005) or for either one subunit of its receptor (Gorelik and Flavell, 2000; Leveen et al., 2002; Li et al., 2006; Marie et al., 2006; Liu et al., 2008) develop a lethal autoimmune syndrome associated with a deficit in Tregs (Fahlen et al., 2005; Marie et al., 2005, 2006; Almeida et al., 2006; Li et al., 2006; Liu et al., 2008). Interestingly, this syndrome is only seen if TGF-β is silenced early in T cell differentiation, suggesting that besides a deficit in tTreg, lymphopenia is an additional trigger of autoimmunity in the absence of TGF-β signaling (Zhang and Bevan, 2012). At the molecular level, TGF-β triggers the binding of Smad3/NFAT complex on the promoter and on the CNS1 enhancer regions of the Foxp3 gene (Tone et al., 2008). However, mice deficient for CNS1 have no alteration in tTreg differentiation (Zheng et al., 2010), suggesting that TGF-β is not required for this process. But mice deficient for both TGF-β and IL-2 are completely deprived of tTregs (Liu et al., 2008), suggesting that TGF-β might compensate a defect in IL-2 and induce Foxp3 expression. How and when IL-2 and TGF-β signaling pathways intersect in the thymus to generate Foxp3^+^ cells remains to be fully elucidated.

Very few studies have focused on the potential role of TGF-β on tTreg homeostasis and function. In the previously cited study from the group of Alexander Rudensky (Marie et al., 2005), the TGF-β1 deficient mice that presented reduced frequencies of CD4^+^CD25^+^ cells, also showed reduced Foxp3 expression among these cells. In addition, Treg deficient for the TGF-βRII showed decreased suppressive function *in vitro*. However, this early study used total CD4^+^CD25^+^ peripheral cells as Tregs, which contains both tTregs and pTregs. Thus, it was impossible at the time to exclude that the effect came from a specific impact of TGF-β on the pTreg subset. More recently, the same group clarified the situation. Indeed, they showed that tTregs purified from CNS1-deficient mice, which present altered TGF-β signaling and lack pTreg differentiation (Zheng et al., 2010; Josefowicz et al., 2012a), did not present altered suppressive function or decreased Foxp3 expression (Josefowicz et al., 2012a). Taken together, these studies suggested that TGF-β is not a main player in tTreg homeostasis or maintenance of Foxp3 expression and suppressive function in these cells.

### TNF-α seems to participate to tTreg development and has a controversial role on tTreg function

TNF-α, a pleiotropic cytokine well known for its major role in the initiation and orchestration of the pro-inflammatory immune response, may also display anti-inflammatory effects (Jacob and McDevitt, 1988; Yang et al., 1994). Mechanistically, TNF-α signals through two different receptors: TNFR-1 and TNFR-2. TNFR-1 is ubiquitously expressed and can induce apoptosis through its intracellular death domain. Furthermore, TNF-α signaling through TNFR-1 under normal conditions leads to activation of the canonical NF-κB pathway that regulates cell activation and differentiation (Chen and Goeddel, 2002; Sun, 2011). On the contrary, TNFR-2 expression is more restrained. This receptor does not have an intracellular death domain and rather induces T cell activation and proliferation (Grell et al., 1998) through the non-canonical NF-κB pathway (Sun, 2011). Furthermore, TNF-α receptors are differentially expressed in Teffs and Tregs. In mice and men, Teff can rapidly upregulate TNFR-2 expression after TCR stimulation, but at the steady state only a very small proportion of Teffs expresses TNFR-2 (Valencia et al., 2006; Chen et al., 2007). In contrast, a significant proportion of Tregs expresses TNFR-2 at the steady state and can further increase its expression upon TCR stimulation or TNF-α incubation (Valencia et al., 2006; Chen et al., 2007). Hence, the dichotomic effect of TNF-α has been – at least in part – attributed to a pro-inflammatory action mediated by TNFR-1 on Teff cells and an anti-inflammatory effect mediated by TNFR-2 signaling on Tregs. However, although different biological functions can be assigned to the signals induced by each of the two receptors, confounding issues come from the existence of receptor crosstalk and shared actions, which are dependent on cell intrinsic and extrinsic factors (Faustman and Davis, 2010).

A likely role for TNF-α on tTreg development comes from TNFR-2 KO or TNFR-2 ligands-deficient (TNF-α/LT-α/LT-β) triple KO mice that show a decrease of Tregs in the thymus (Chen et al., 2013). These results need however to be interpreted with caution since lymphopenia *per se* or alterations of the thymic stromal architecture might affect tTreg generation. Further elucidation of the role of TNF-α and TNF receptors awaits the generation of mice with conditional ablation of TNF receptors in the Treg lineage.

Data on the effect of TNF-α on Treg function are fragmented and sometimes controversial. *In vitro*, TNF-α through TNFR-2 signaling increases mouse tTreg proliferation in the presence of IL-2 (Chen et al., 2007) and optimally activates Tregs increasing the expression of receptors of the TNF super family, such as TNFR-2, 4-1BB, and OX40 (Hamano et al., 2011). In addition, pre-incubation of Tregs with TNF-α can improve their *in vitro* suppressive function (Chen et al., 2007). However, Tregs obtained from WT or TNFR-2 KO mice appear to have similar *in vitro* suppressive activity (van Mierlo et al., 2008). Moreover, and at odds with mice results, TNF-α seems to reduce Treg suppressive function in humans (Valencia et al., 2006; Chen et al., 2007; Nagar et al., 2010; Zanin-Zhorov et al., 2010). The main issue of these experiments is that TNF-α can act on both Treg and Teff populations and that activated Teffs, which express TNFR-2, are less sensitive to Treg-mediated suppression (Chen and Oppenheim, 2010).

*In vivo*, TNF-α seems to contribute to Treg homeostasis (Chen et al., 2013; Chopra et al., 2013) and one paper implicates TNFR-2 and the non-canonical NF-kB pathway in this action (Rauert et al., 2010). Also, TNF-α seems to enhance Treg function *in vivo*, as suggested by our own results showing that activated Teffs can boost Treg proliferation and suppressive function, partly by a TNF-α-mediated mechanism probably implying the non-canonical NF-kB pathway (Grinberg-Bleyer et al., 2010b). Additionally, TNF-α could improve Treg proliferation and accumulation in mouse models of cecal ligation puncture, colitis, and cancer (Chen et al., 2007, 2013; Chopra et al., 2013). Moreover, the fact that TNF-α^−*/*−^LTα^−*/*−^LTβ^−*/*−^ and TNFR-2^−*/*−^ mice possess less Tregs in the periphery supports the idea that TNF-α, like IL-2, plays a role in Treg homeostasis (Chen et al., 2013; Chopra et al., 2013). However, neither TNF-α^−*/*−^ nor TNFR-2^−*/*−^ mice develop spontaneous autoimmunity and Tregs recovered from these mice express the same level of Foxp3 (Chen et al., 2013). Nevertheless, as the lack of TNFR-2 expression on Teffs could impact their pathogenicity, it is difficult to evaluate the individual contribution of the TNF-α/TNFR-2 pathway to Treg and Teff function. Interestingly, recent work using transfer of highly purified WT or TNFR-2 KO Tregs in RAG^−*/*−^ mice suggests that colitis suppression could be dependent on TNFR-2 expression by Tregs *in vivo* (Housley et al., 2011; Chen et al., 2013). Collectively, these data point to an important role of signaling though TNFR-2 in the suppressive function of Tregs *in vivo* and calls for confirmation with the use of Tregs with conditional ablation of TNF receptors.

In humans, TNF-α has been implicated in the physiopathology of autoimmune diseases and consequently anti-TNF-α treatments (antibodies or soluble receptor) have been used with successful results obtained in Crohn’s disease and rheumatoid arthritis (Chan and Carter, 2010). Interestingly, beyond dampening TNF-α’s pro-inflammatory effect, anti-TNF-α treatment has been associated with an accumulation of Tregs (Ehrenstein et al., 2004) and with improved Treg function (Ehrenstein et al., 2004; Valencia et al., 2006; Nadkarni et al., 2007). Likewise, a recent study showed that TNF-α present in the synovial fluid of RA patients reduced Treg suppressive function and this function was restored in anti-TNF-α treated patients (Nie et al., 2013). However, increased frequencies of Tregs, could be alternatively explained by a relative reduction of the activated Foxp3^−^ cells without a direct change in Treg homeostasis. Indeed, in mice transgenic for human TNF-α, which develop spontaneous arthritis, both pools of Teffs and Tregs decrease during the disease course and increase during anti-TNF-α treatment (Biton et al., 2011). Of note, not all patients respond to anti-TNF therapies and even some of them paradoxically develop anti-TNF-induced autoimmune diseases such as multiple sclerosis (MS), T1D, inflammatory bowel disease, vasculitis, lupus, and many others (Perez-Alvarez et al., 2013) suggesting that TNF-α could also have a regulatory role in other human autoimmune conditions. Although the underlying cause is not yet understood, some insight for the unexpected role of TNF-α in T1D and MS may be gained from the corresponding murine models. Indeed, TNF-α can exacerbate T1D and EAE when administered early during disease initiation and can inhibit disease progression when administered at later time points (Ruddle et al., 1990; Willenborg et al., 1995; Wu et al., 2002). Therefore, it is possible that the opposite response to anti-TNF therapies originates from the opposite roles that TNF may have during the different phases of the disease.

## Peripherally Derived-Regulatory T Cells

The group of J. Lafaille was among the first to described pTregs in 2002. They showed that the transfer of CD4^+^ spleen T cells could prevent EAE in an IL-2-dependent process involving the differentiation of CD4^+^CD25^+^ Tregs from CD4^+^CD25^−^ T cell precursors (Furtado et al., 2002). Induced from naïve CD4^+^ T cells in the periphery, pTregs present a distinct and broader TCR repertoire than tTreg (Haribhai et al., 2011; Josefowicz et al., 2012b). Indeed, pTreg differentiation mainly occurs in the context of bacterial or viral infection (Robertson et al., 2006; Curotto de Lafaille and Lafaille, 2009; Ertelt et al., 2009), in tumors (Nishikawa et al., 2003; Liu et al., 2007), or in mucosal tissues notably in a context of oral tolerance (Mucida et al., 2005; Coombes et al., 2007; Sun et al., 2007; Josefowicz et al., 2012a). A recent study suggested that pTreg main function would be the prevention of mucosal Th2-mediated immunity, notably in the gastrointestinal tract and lungs (Josefowicz et al., 2012a). pTregs would also be involved in the induction of tolerance to commensal microbiota (Sun et al., 2007; Josefowicz et al., 2012a).

The differentiation of pTregs requires antigenic stimulation in a defined anti-inflammatory environment, process orchestrated in part by dendritic cells (DC). Several DC subsets have been associated with pTreg induction (Yamazaki et al., 2007), including plasmacytoid DC (Ochando et al., 2006; Goubier et al., 2008) and CD103^+^ DCs, which following education by retinoic acid (RA) differentiate into tolerogenic DC, mainly in the intestine (Coombes et al., 2007; Sun et al., 2007). H. Von Boehmer’s group showed that targeting the antigen toward DCs through DEC205 recognition induced the conversion of CD4^+^ naïve T cells into CD4^+^CD25^+^Foxp3^+^ pTreg (Kretschmer et al., 2005). This induction was shown to be dependent on TGF-β (Yamazaki et al., 2008). Indeed, mice deficient for TGF-β in Langerhans cells (a specialized subset of DC in the skin) develop signs of skin auto immunity (Kaplan et al., 2007), in agreement with the hypothesis that a lack of DC in the skin may lead to local auto-immunity due to a defect in Treg. The role of IL-2 or TNF-α produced by DC in the induction of pTreg remains to be fully elucidated.

### TGF-β is the master regulator of pTreg differentiation and homeostasis

The differentiation of pTregs from CD4^+^CD25^−^Foxp3^−^naive T cells requires TGF-β. Initial studies showed that *in vitro* treatment of murine or human CD4^+^CD25^−^Foxp3^−^naive T cells with TGF-β induced Foxp3 expression in these cells and conferred a suppressive function (Chen et al., 2003; Walker et al., 2003). Then, a prominent role for TGF-β in pTreg differentiation has been demonstrated *in vivo* (Marie et al., 2005, 2006; Coombes et al., 2007; Sun et al., 2007), notably in the context of oral tolerance (Curotto de Lafaille and Lafaille, 2009). In addition, mice deficient for the TGF-β-sensitive CNS1 enhancer do not present any differentiation of pTregs (Zheng et al., 2010; Josefowicz et al., 2012a) confirming the essential role of TGF-β in pTreg induction. Interestingly, *in vivo* pTregs differentiation by TGF-β can occur both in physiological (Coombes et al., 2007; Sun et al., 2007) and pathological (Grainger et al., 2010) situations.

Two kinds of regulators of pTreg differentiation can be distinguished. First, those that interfere with TGF-β signaling like TNF-α (Zhang and Bevan, 2012), or those that induce PI3K signaling. Of importance, the PI3K/Akt/mTOR pathway is a strong inhibitor of Foxp3 expression, which prevents both tTreg and pTreg differentiation (Haxhinasto et al., 2008; Sauer et al., 2008). Consistent with these observations, a very recent study demonstrated that pTreg differentiation could not occur in presence of the C3a or C5a signaling pathway. This pathway blocks Foxp3 expression by induction of PI3K signaling and repression of TGF-β expression (Strainic et al., 2013). Second, pTreg differentiation can be improved by molecules that increase TGF-β signaling (Xu et al., 2010), like CTLA-4 (Karman et al., 2012). Thus, modulating TGF-β or PI3K signaling in the periphery might represent a promising approach to tip the balance in favor of pTreg.

Of importance, TGF-β has a dual and opposite role in the immune system: it participates not only in the generation of pTregs, but also, in conjunction with IL-6 or IL-4, induces the differentiation of pro-inflammatory Th17 cells or Th9 cells, respectively (Jabeen and Kaplan, 2012; Maddur et al., 2012). Consequently, TGF-β administration could aggravate inflammation and autoimmunity. Moreover, TGF-β pleiotropic effects outside the immune system and toxicity limit its therapeutic application (Flanders and Roberts, 2001) and systemic administration of TGF-β has been rapidly abandoned by the pharmaceutical industry (Prud’Homme, 2007).

### IL-2 signaling is mandatory for pTreg differentiation, homeostasis and stability

Although IL-2 by itself is not sufficient to generate pTregs, it seems to be critical for the development of functional CD4^+^CD25^+^Foxp3^+^ pTregs induced by TGF-β (Chen et al., 2003; Davidson et al., 2007; Zheng et al., 2007). Indeed, addition of a neutralizing anti-IL-2 antibody to the culture strikingly abolishes the induction of Foxp3, and IL-2^−*/*−^ or Stat5^−*/*−^ naïve T cells are unable to generate pTregs (Davidson et al., 2007; Laurence et al., 2007; Yao et al., 2007; Zheng et al., 2007). Interestingly, the role of IL-2 is non-redundant, as other common gamma chain receptor using cytokines cannot restore pTreg generation in IL-2^−*/*−^ T cells (Davidson et al., 2007; Zheng et al., 2007). Likewise, the group of D. Horwitz reported that IL-2 also potentiates pTreg suppressive function and expression of key Treg-signature molecules such as CTLA-4, GITR, and CD122 (Zheng et al., 2007). Different mechanisms have been proposed to explain IL-2 action in the TGF-β mediated induction system: sustained Foxp3 expression via JAK3/STAT5 signaling (Chen et al., 2011), enhancement of proliferation and survival of newly generated pTregs, or increased conversion of latent to active TGF-β via the urokinase receptor pathway (Nykjaer et al., 1994; Odekon et al., 1994).

In the case of lymphopenic recipients, the generation of pTregs upon transfer of naive T cells seems dependent on IL-2 production by activated T cells (Knoechel et al., 2005). Along these lines, low-dose IL-2 injection into irradiated recipients of allogeneic T cells increases the generation of donor-derived pTregs (Shin et al., 2011). However, the specific contribution of tTregs and pTregs to the Treg increase induced by exogenous IL-2 administration to lympho-replete mice is still unexplored (Tang et al., 2008; Webster et al., 2009; Grinberg-Bleyer et al., 2010a).

**Figure 2:**
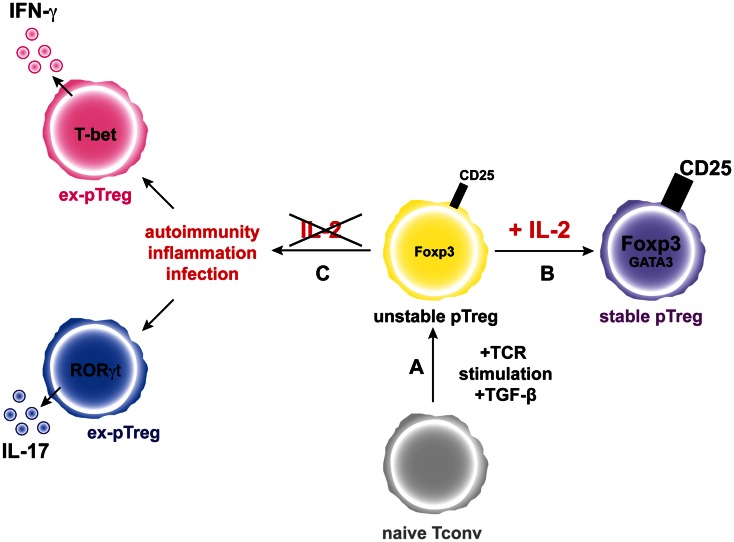
**IL-2 favors the generation and increases the stability of the pTreg phenotype**. **(A)** Activation of a naive conventional T cells through TCR stimulation and in the presence of TGF-β leads to generation of unstable pTregs that express moderate levels of Foxp3 and CD25. **(B)** In the presence of IL-2, pTregs increase the expression levels of Foxp3 and CD25 and the pTreg phenotype is stabilized. **(C)** During inflammation and in the absence of IL-2, the pTregs convert into “ex-pTregs” that can produce different pro-inflammatory cytokines, such as IFN-γ or IL-17 depending on the environmental context.

*In vitro* and *in vivo* recently differentiated pTregs present unstable Foxp3 expression (Floess et al., 2007; Miyao et al., 2012), which can be lost in an inflammatory context, giving rise to “ex-Tregs” producing pro-inflammatory cytokines (Zhou et al., 2009). Importantly, in most of the studies describing Foxp3 instability, Tregs were put in an environment lacking IL-2 (Tang et al., 2008; Duarte et al., 2009; Oldenhove et al., 2009; Zhou et al., 2009). However, IL-2 administration could prevent the conversion of Tregs (Tang et al., 2008; Duarte et al., 2009; Oldenhove et al., 2009; Zhou et al., 2009). Furthermore, unstable pTregs are exclusively located in the CD4^+^CD25^−^Foxp3^*low*^ compartment and, upon time and “education,” they acquire increased CD25 and Foxp3 expression as well as a demethylated CNS2 – a well known marker of Treg stability (Floess et al., 2007; Polansky et al., 2008; Komatsu et al., 2009; Miyao et al., 2012). Also, IL-2 could prevent conversion of recently differentiated pTregs by the induction and regulation of GATA-3 expression (Wang et al., 2011; Wohlfert et al., 2011). Taken together, all these studies suggest that IL-2 plays an important role in the “education” of pTregs (Figure 2). However, further studies are required to determine the additional factors involved in the stability of the Treg lineage.

### The emerging role of TNF-α in pTreg homeostasis and function

Interestingly, Housley et al. (2011) point out that pTregs, contrary to tTregs, may not require TNFR-2 expression to suppress *in vivo*. However, TGF-β pre-incubation can render tTregs from TNFR-2^−*/*−^ mice as efficient as WT cells, suggesting that in this study, the implication of TNFR-2 in the suppressive capacity of pTregs may be hidden by the TGF-β pre-incubation (Housley et al., 2011). Consequently, the role of TNFR-2 in the suppressive function of pTregs is still unclear.

In humans, the above-described observation that blocking TNF-α is associated with an increase of Tregs (Ehrenstein et al., 2004) can alternatively be explained by the fact that TNF-α exposure could hamper pTreg induction. Indeed, it is not clear whether the accumulated Tregs observed after anti-TNF-α treatment are tTreg or pTreg. Then, it could be possible that TNF-α may have a negative effect on the induction of pTreg cells that would be removed during anti-TNF-α administration. Indeed, it was shown that blocking TNF-α increased susceptibility to *Histoplasma capsulatum* infection and induced a population of CD4^+^CD25^+^ T cells possessing IL-10-dependent suppressive function in mice (Deepe and Gibbons, 2008). Besides, TNF-α can inhibit TGF-β-driven pTreg induction from Foxp3^−^ Teffs in an EAE model (Zhang et al., 2013). Finally, it has been pointed out that soluble TNF-α and membrane-bound TNF-α may differently affect the process of pTreg induction *in vitro* (Kleijwegt et al., 2010). A better knowledge of the mechanisms ruling expression of TNFR-1 and TNFR-2 on tTregs and pTregs could help explain potential different effects of TNF-α on these two cell populations.

## Concluding Remarks

We focused our review on three major cytokines that regulate different aspects of Treg biology, namely IL-2, TGF-β, and TNF-α, because they are of great fundamental and clinical importance. Indeed, immunotherapies based on a better knowledge of the impact of cytokines on Treg biology are emerging (Chan and Carter, 2010; Koreth et al., 2011; Saadoun et al., 2011). However, we need to better understand the division of labor of pTreg and tTreg in the fine tuning of the immune response. It is not yet clear if each cell type acts preferentially at different localizations, at different timepoints during the initiation, expansion, contraction, and memory generation of the immune response, or has specific targets for suppression. Also, we need to dissect the specific cytokine requirements for tTreg and pTreg generation, homeostasis and function, which are sometimes distinct and sometimes shared. Only with that information at hands will the extraordinary promises of tTregs and pTregs be fully exploited in safe and effective clinical applications.

## Conflict of Interest Statement

The authors declare that the research was conducted in the absence of any commercial or financial relationships that could be construed as a potential conflict of interest.
